# Supplementary material for: Altered thymic differentiation and modulation of arthritis by invariant NKT cells expressing mutant ZAP70
Source: Nat Commun. 2018 Jul 6;9:2627. doi: 10.1038/s41467-018-05095-7 (PMC6035278; doi:10.1038/s41467-018-05095-7)
Supplement: Supplementary file 1 — Supplementary Information [file 41467_2018_5095_MOESM1_ESM.pdf]

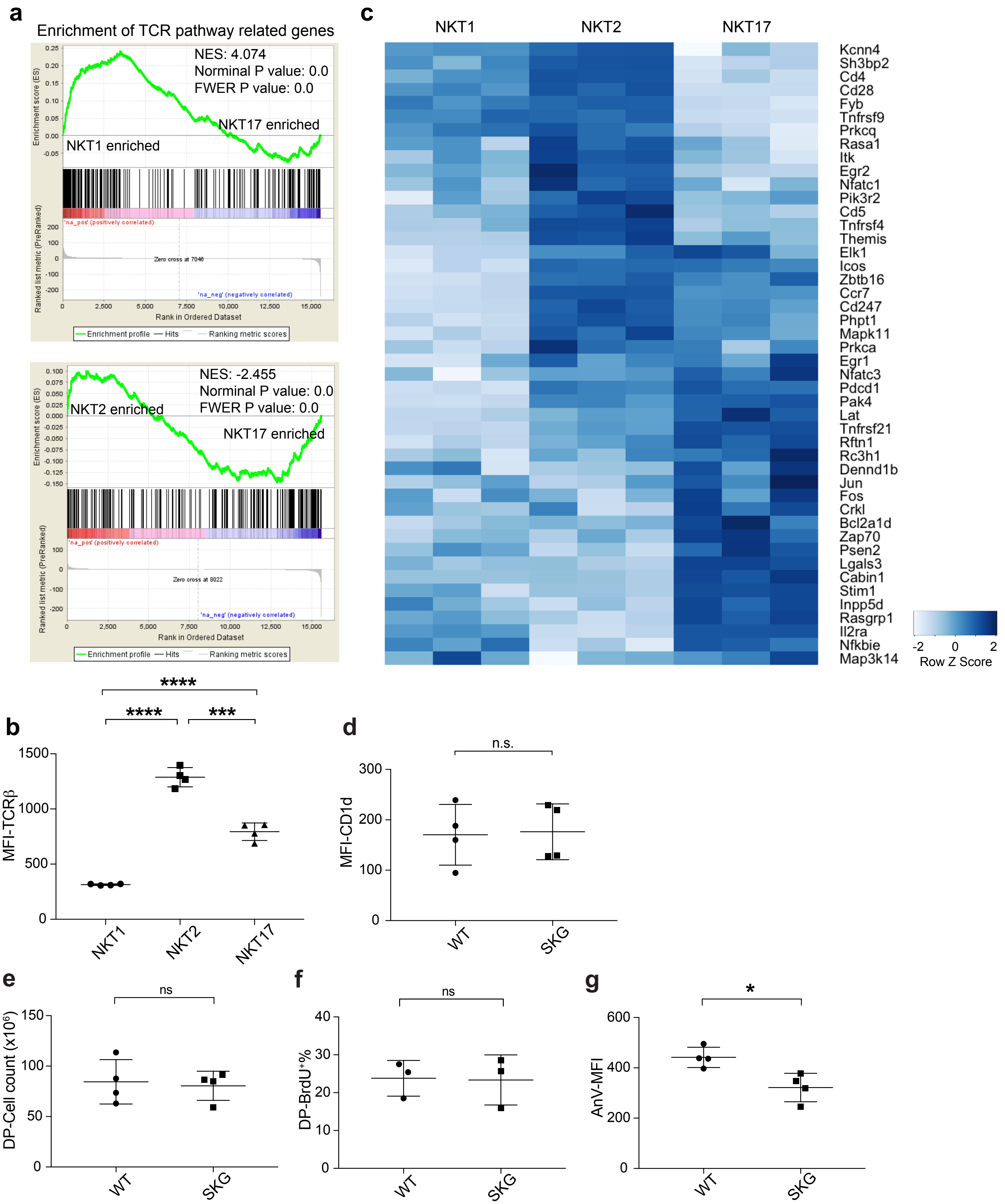

Supplemental Figure 1. Enrichment of TCR signaling related genes in iNKT cell subsets and comparison of DP cells in WT and SKG mice (related to Figure 1.)

(a) GSEA showing the enrichment of TCR signaling pathway related genes in iNKT cell subsets of BALB/c thymus, related to Figure 1a.
(b) Expression level of TCRβ in iNKT cell thymic subsets (c) Differentially expressed TCR pathway genes in NKT2 and NKT17 cells obtained from triplicate RNA-seq runs are shown in a heatmap. (d) Expression of CD1d in CD4 and CD8 double positive (DP) thymocytes in adult WT and SKG mice. (e) Absolute numbers of DP cells from WT or SKG mice. (f) Incorporation of BrdU in DP cells examined 20h after injection. (g) Expression of AnnexinV (AnV) in WT and SKG DP cells. (b-g) Data are representative of three independent experiments. Graphs represent mean ± SD with symbols representing individual mice.*p<0.05; **p<0.01; ***p<0.001; ****p<0.0001; n.s. not significant (unpaired two-tailed Student's t test)

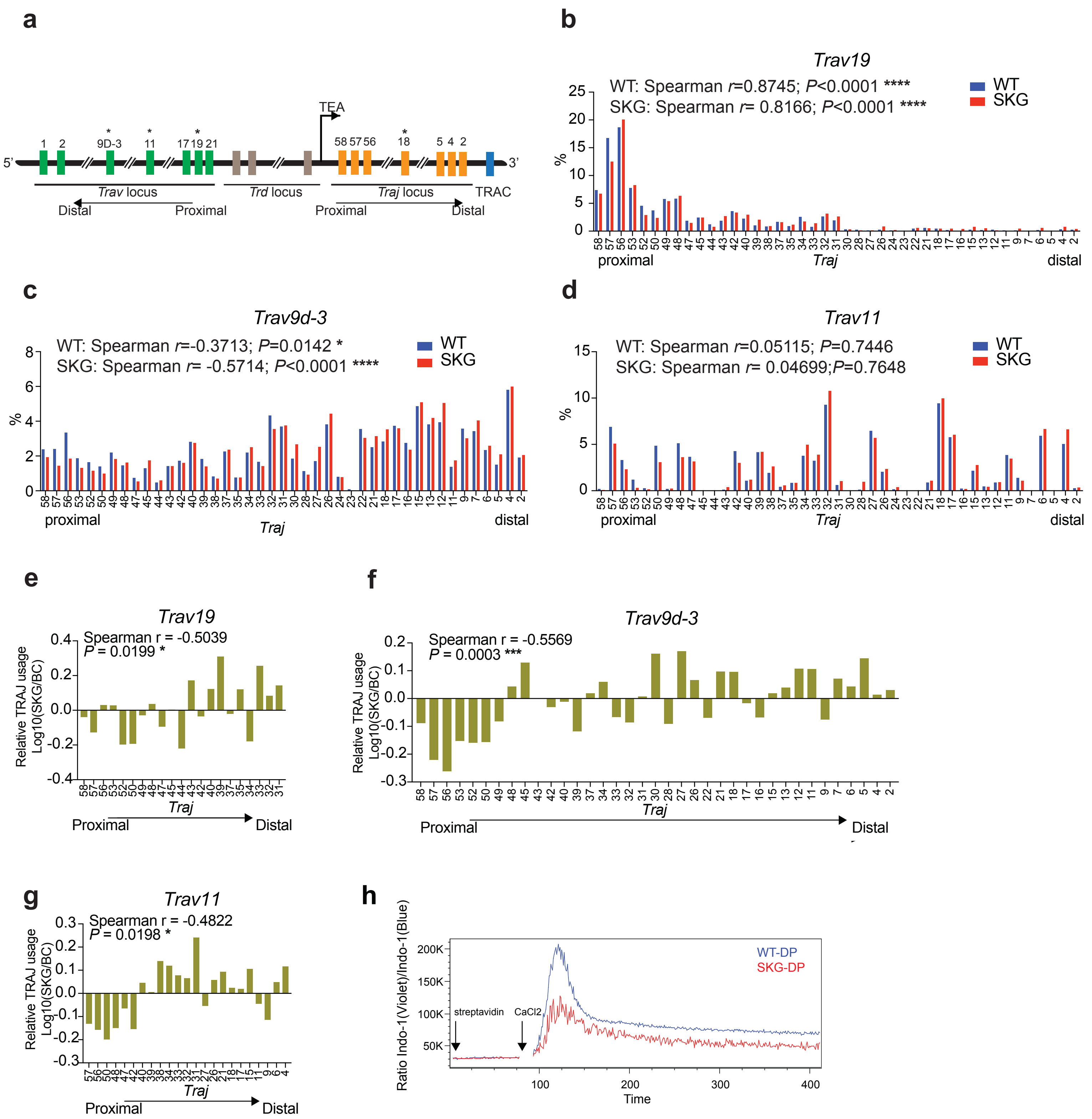

Supplemental Figure 2. Comparison of TCR repertoires in WT and SKG DP cells
(a) Representation of the *TCRαδ* locus. Some functional Trav gene segments shown in green, and *Traj* gene segments in orange.
T early activation (TEA) controlling primary *Trav-Traj* rearrangements of 3' *Trav* (proximal) and 5' *Traj* (proximal) segments are also shown.
(Modified from: http://www.imgt.org/textes/IMGTrepertoire/LocusGenes/index.php?repertoire=locus&species=mouse&group=TRA)
(b-d) *Traj* usage by *Trav19* (b), *Trav9d-3* (c) and *Trav11*(d) in WT and SKG pre-selection DP ($CD69^{-}TCR\beta^{-}$) cells was determined as a percentage of total Traj gene reads for each Trav gene. (e-g) Relative *Traj* (with usage higher than 1% in each case) usage was calculated as a ratio of *Traj* gene usage in SKG thymocytes to WT. All Traj genes displayed from 5' (proximal) to 3' (distal) along the genomic locus. Ranked Spearman correlation coefficient was calculated on log-transformed values of ratios; P value of correlation shown.
(h) Flow cytometry of double positive thymocytes from WT and SKG mice loaded with the fluorescent Ca^{2+} indicator Indo-1.
Arrows indicate the additions of free streptavidin (2ug/ml) and $CaCl_2$ (5mM).

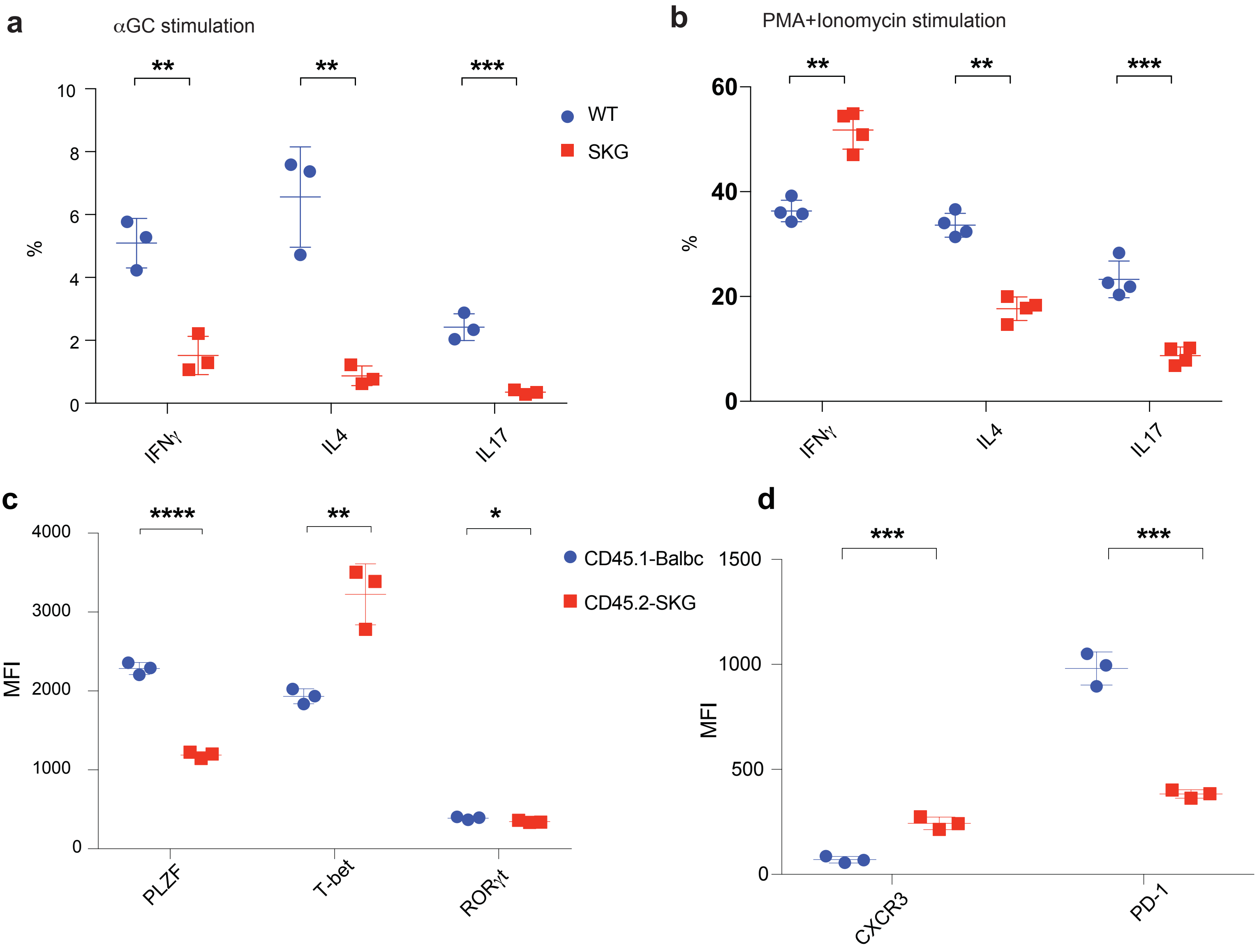

Supplemental Figure 3. *in vitro* activation of thymic iNKT cells and mixed bone marrow chimera
(a, b) Thymocytes were enriched for iNKT cells by depleting CD8^{+} cells, and stimulated *ex vivo* on a CD1d coated plate with αGC (a) or PMA and ionomycin (b). Cytokine production by WT and SKG iNKT cells were examined. (c, d) Thymic iNKT cells from mixed bone marrow chimeric recipient mice were separated into WT (CD45.1) and SKG (CD45.2) and the expression of transcription factors (c) and surface markers (d) were determined. Data are representative of three independent experiments. Graphs represent mean ± SD with symbols representing individual mice.*p<0.05; **p<0.01; ***p<0.001; ****p<0.0001; n.s. not significant (unpaired two-tailed Student's t test)

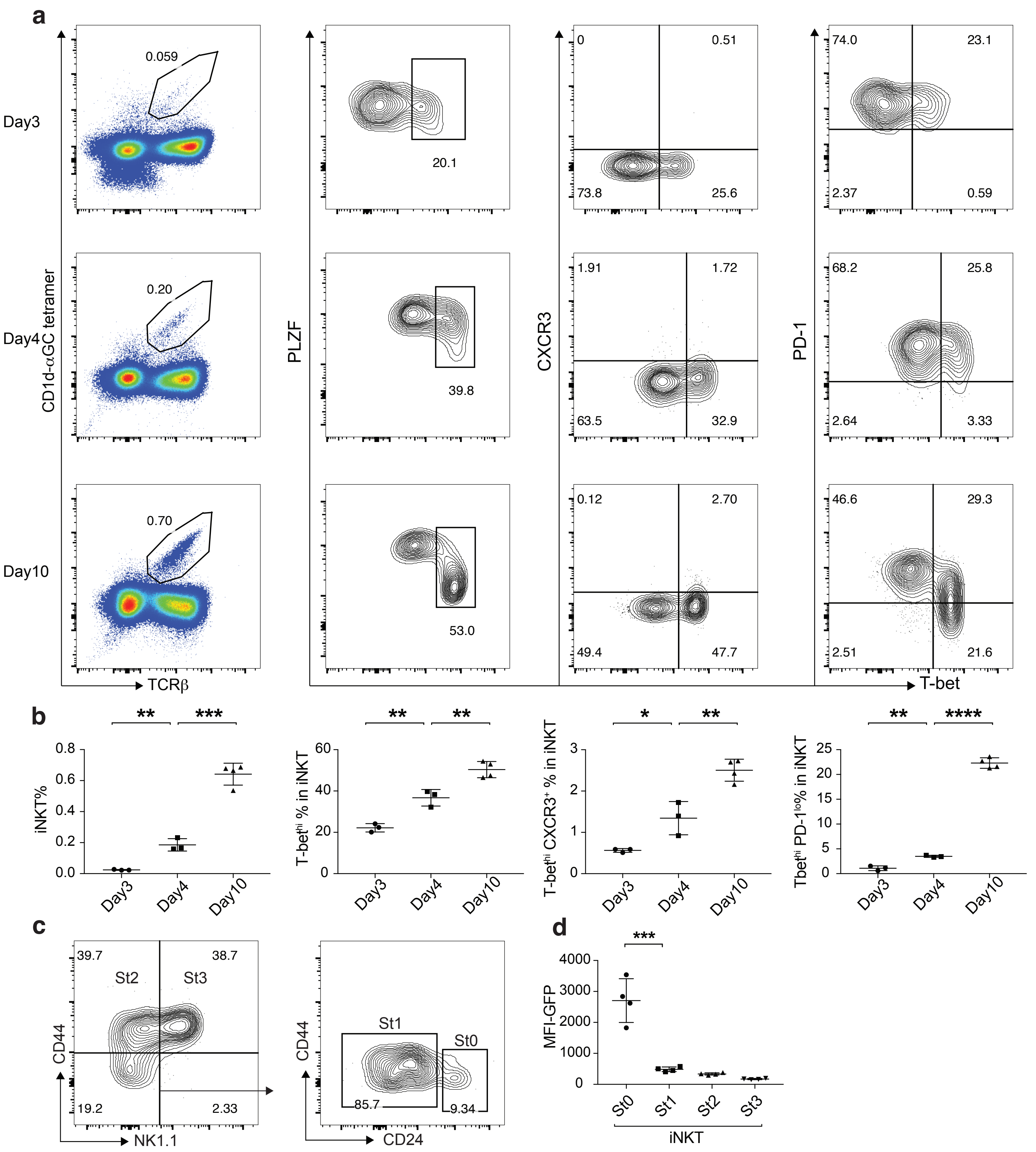

Supplemental Figure 4. Ontogeny of iNKT cells in BALB/c mice and thymic organ culture of Nur77^{GFP} mice
(a) BALB/c mice at 3 day, 4 day or 10 day of age were examined for thymic iNKT cells. Percentage of iNKT cells among CD8$^-$ thymocytes, T-bet-^{hi} population in total iNKT cells and expression of CXCR3 and PD-1 were shown as in Figure 2. (b) Compiled data of (a).
(c, d) Thymic lobes from day 0 C57BL/6 or *Nur77^{gfp}* mice were cultured as in Figure 2 for 5 days, and iNKT cells were divided first by the expression of CD44 and NKT1.1 into Stage2 (St2, CD44^{hi} NK1.1$^-$) and Stage3 (St3, CD44^{hi} NK1.1$^+$). CD44$^-$NK1.1$^-$ cells were further separated by CD24 expression into Stage0 (CD24^{hi}) and Stage1(CD24^{lo}). Expression of GFP was compared among the different stages (d). Data are representative of three independent experiments. Graphs represent mean ± SD with symbols representing individual mice (or thymic lobe in d.). *p<0.05; **p<0.01; ***p<0.001; ****p<0.0001; n.s. not significant (unpaired two-tailed Student's t test).

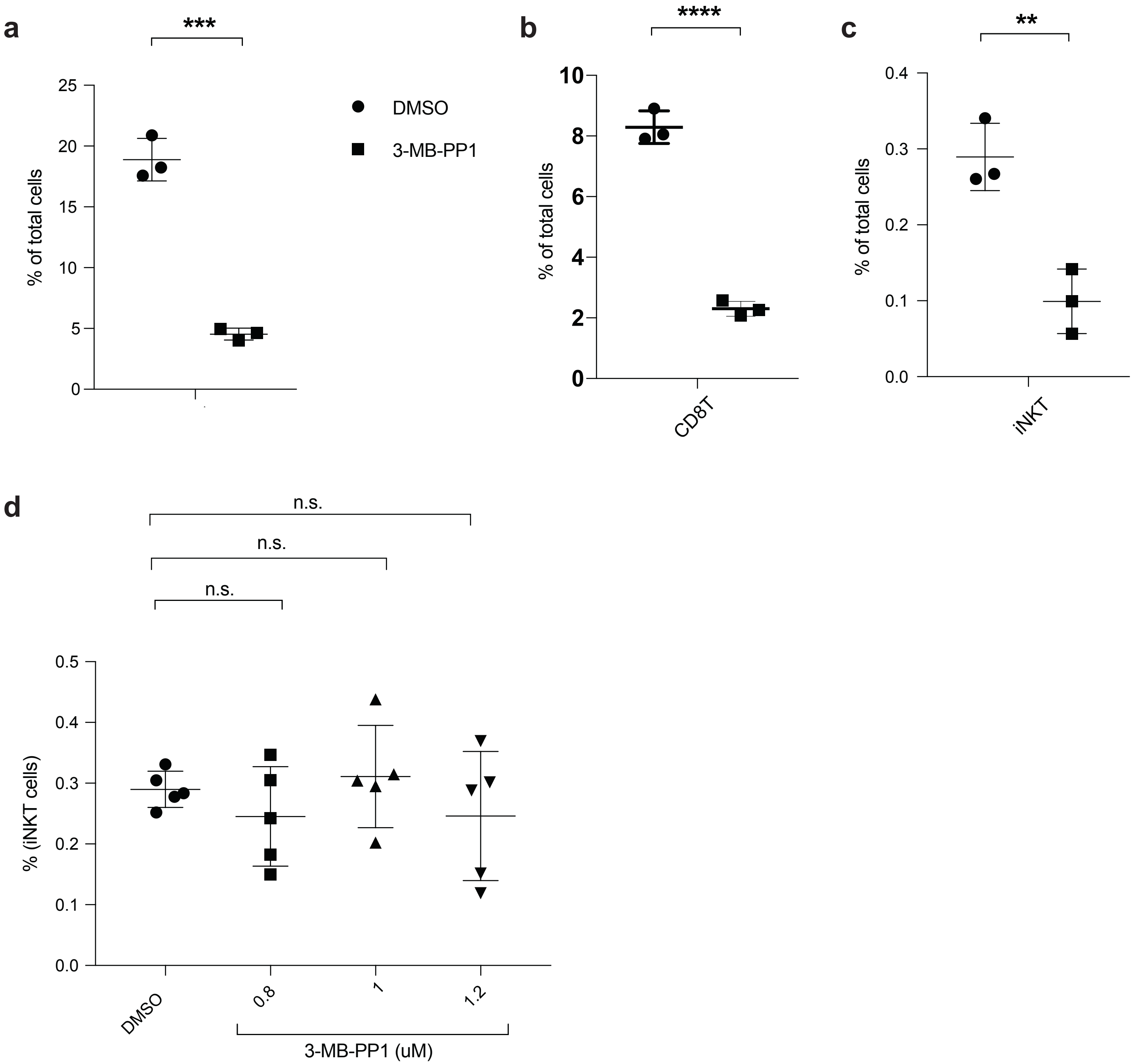

Supplemental Figure 5. T cells from ZAP70 AS mice in organ culture (related to Figure 3).
(a-c) Day 0 thymic lobes from ZAP70AS mice were cultured as in Figure 3. 1uM of the inhibitor 3-MB-PP1 was added at the start of culture, and CD4T, CD8T and iNKT cells were examined on day 7. (d) Day 0 thymic lobes from ZAP70AS mice were cultured as in Figure 3. Different doses of the inhibitor 3-MB-PP1 was added on the second day of culture, and iNKT cells were examined on day 7.
Data are representative of three independent experiments.Graphs represent mean ± SD with symbols representing individual thymus lobe.
**p<0.01; ***p<0.001; ****p<0.0001; n.s. not significant (unpaired two-tailed Student’s t test)

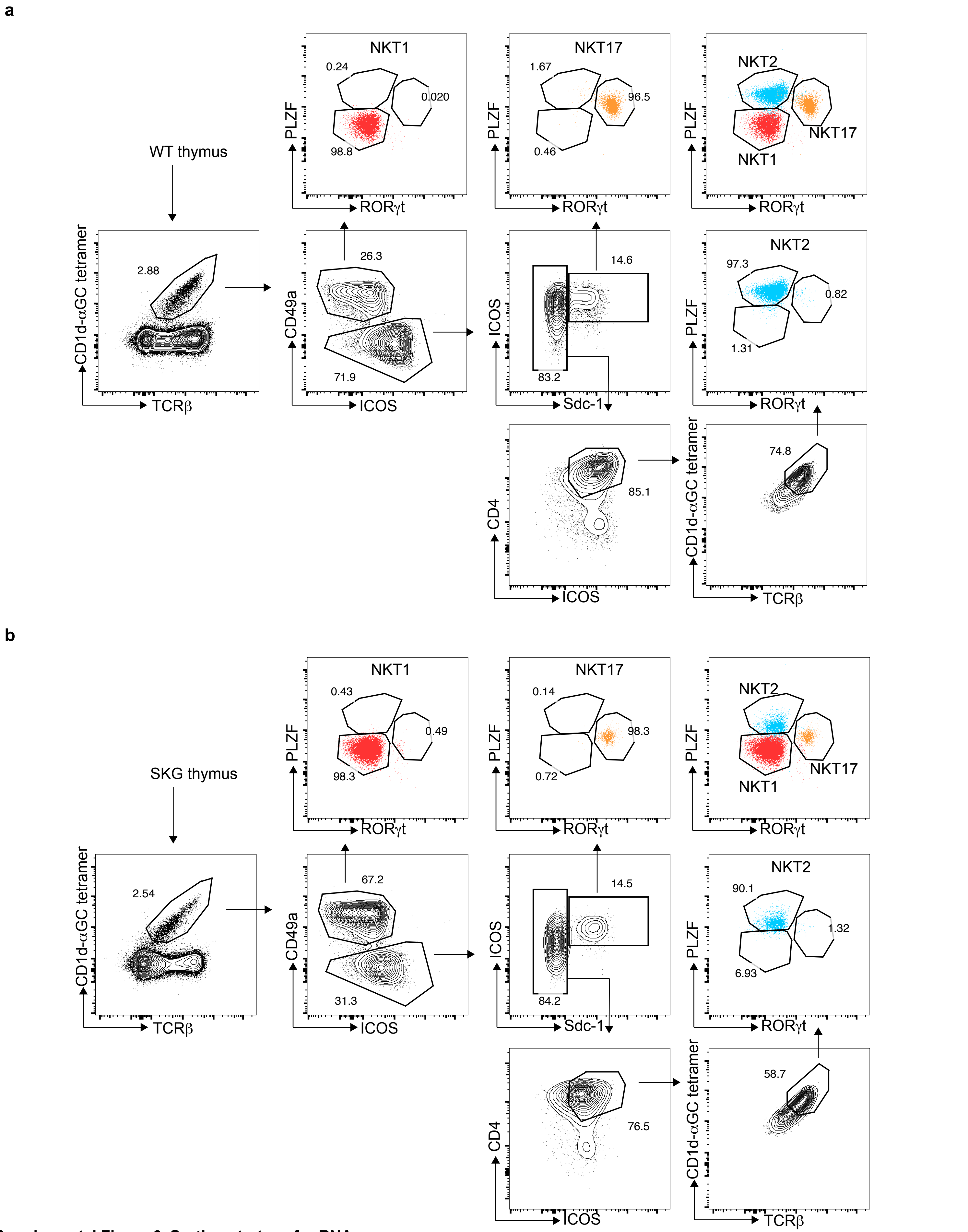

Supplemental Figure 6. Sorting strategy for RNA-seq

(a) Thymic iNKT cells from 8-week-old WT mice were gated as TCRβ^{int} αGC tetramer^{+} in live CD8^{-} cells. Total iNKT cells were separated into NKT1 (CD49a^{+}ICOS^{lo}), NKT2 (CD49a^{-}Sdc-1^{-}ICOS^{hi}CD4^{+}TCRβ^{hi}) and NKT17 (CD49a^{-}ICOS^{hi}Sdc-1^{+}). Purity of each subset was demonstrated by the expression of the transcription factors PLZF and RORγt. (b) Purity of thymic iNKT cell subsets from SKG mice. Three independent sorts were carried out using age and sex matched WT and SKG mice each time.

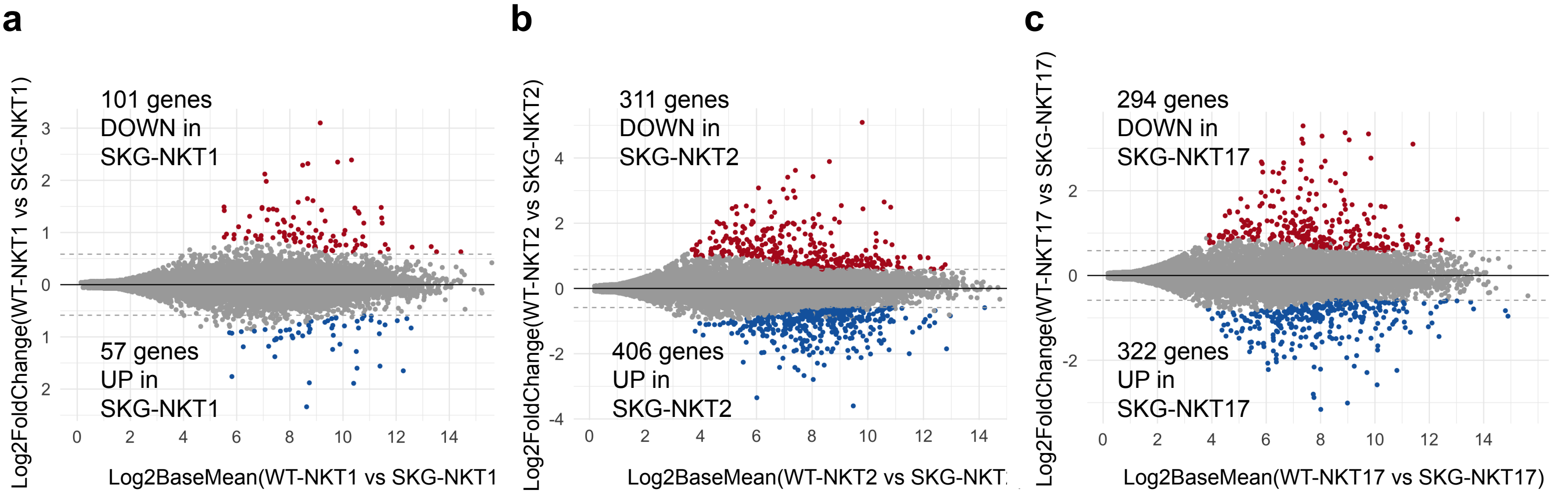

Supplemental Figure 7. Differential gene expression analysis comparing iNKT cell subsets from SKG and WT mice.
(a-c) MA plots showed differentially expressed genes for pairwise comparisons of RNA-seq data from iNKT cell subsets from SKG and WT mice. A fold change cutoff of 1.5 and P-value cut off of 0.1 were applied to color code differentially expressed genes on the plot. The numbers of DE genes were indicated. The RNA-seq data from WT mice are the same data analyzed in Figure 1. For details see Online Methods.

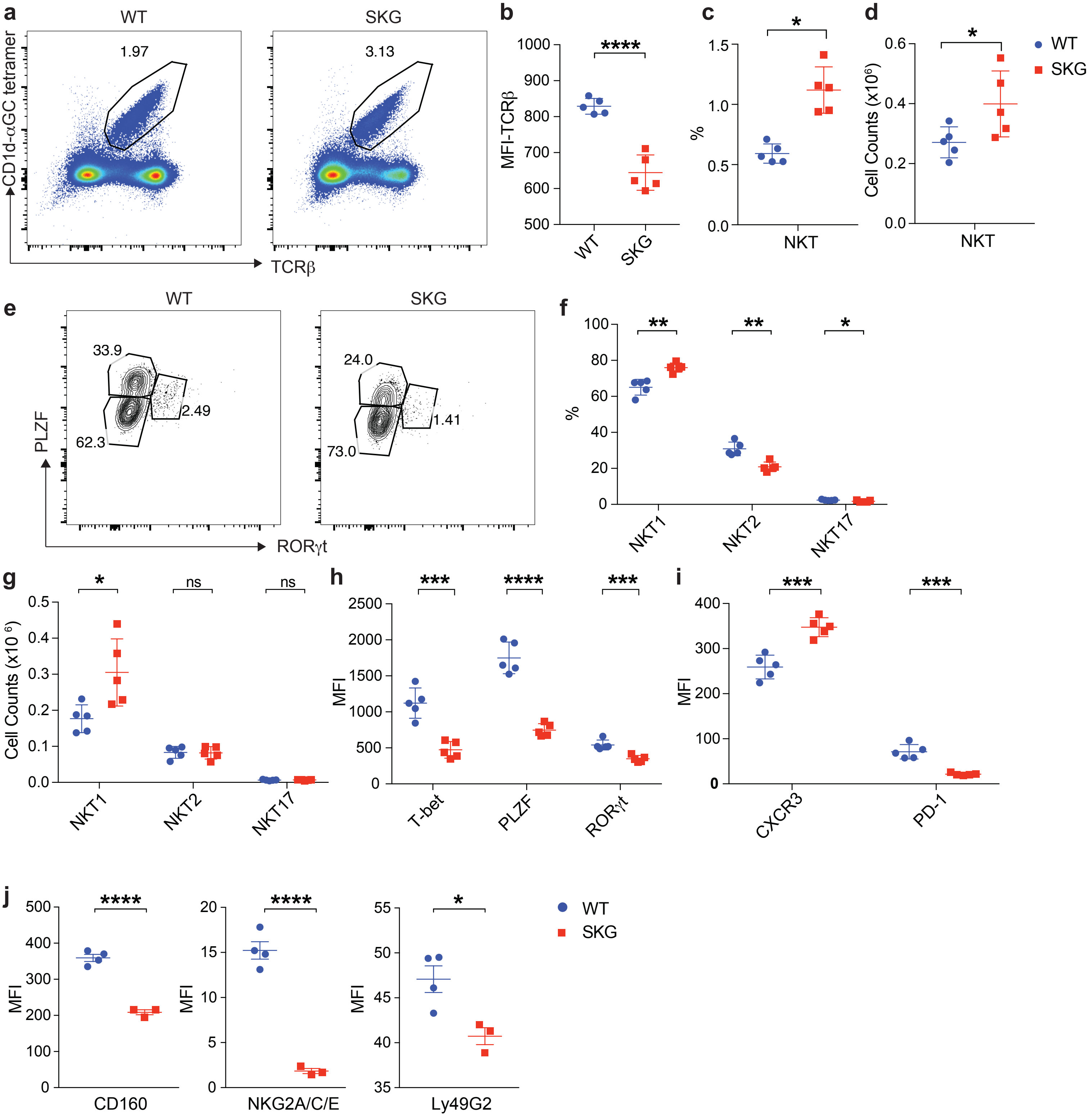

Supplemental Figure 8. Splenic iNKT cell subsets in SKG mice
(a) Splenic iNKT cells were gated as TCRβ^{int} αGC tetramer^{+} in live CD8^{-}CD19^{-} cells. (b) The expression of TCRβ in WT or SKG NKT cells was compared. (c, d) Percentage in live cells (c) and cell numbers (d) of splenic iNKT cells in WT and SKG mice. (e) iNKT cell subsets were gated as PLZF^{lo} RORγt^{-} (NKT1), PLZF^{hi} RORγt^{-} (NKT2) and PLZF^{int}RORγt^{+} (NKT17). (f, g) Percentage in total iNKT cells (f) and absolute numbers (g) of iNKT subsets in WT and SKG mice (h, i) Expression of transcription factors and subset characteristic surface markers in iNKT cells from WT and SKG mice. (j) Expression of CD160, NK2a/c/e and Ly49G2 in splenic iNKT cells from WT and SKG mice. Data are representative of three independent experiments. Graphs represent mean ± SD with symbols representing individual mice. *p<0.05; **p<0.01; ***p<0.001; ****p<0.0001; n.s. not significant (unpaired two-tailed Student's t test).

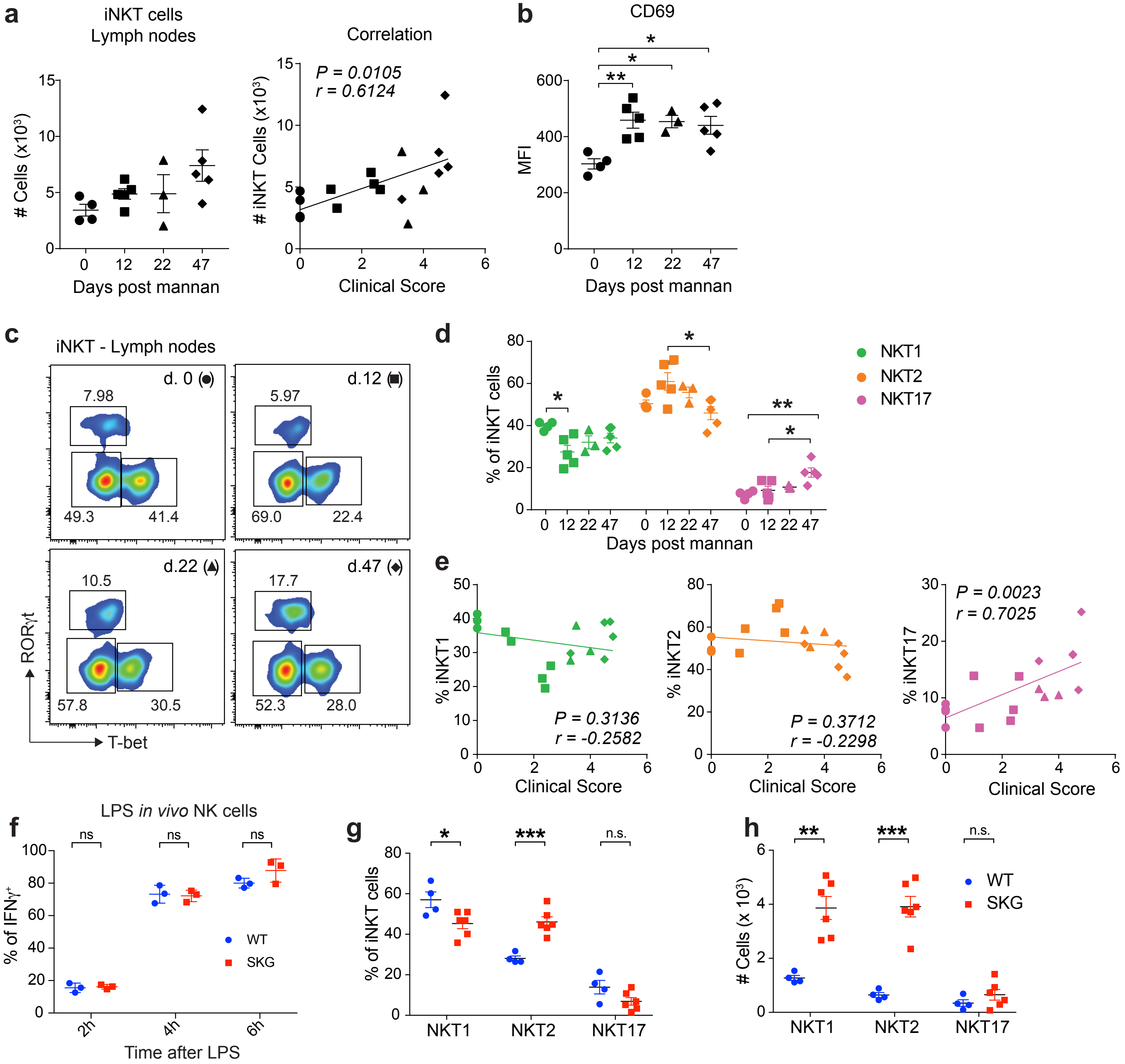

Supplemental Figure 9. Changes of LN iNKT cells during the course of mannan-induced arthritis.
(a) Total number of iNKT cells in joint draining lymph nodes and the correlation with clinical arthritis score. Day 0 (d.0, circle), day 12 (d.12, square), day 22 (d.22, triangle) and day 47 (d.47, diamond) post mannan injection. (b) Expression of CD69 on iNKT cells in joint draining lymph nodes during mannan-induced arthritis in SKG mice. (c) Representative flow cytometry plots showing iNKT ($TCR\beta^{int}$ CD1d-αGC $tetramer^{+}$) cell subsets based on transcription factor staining in joint draining lymph nodes (brachial, axillary and popliteal) during the course of mannan-induced arthritis in SKG mice. (d) Frequency of NKT1 ($T\text{-}bet^{+}ROR\gamma t^{-}$, left, green),NKT2 ($T\text{-}bet^{-}ROR\gamma t^{-}$, middle, orange) and NKT17 ($T\text{-}bet^{-}ROR\gamma t^{+}$, right, purple) in total iNKT cells from joint draining lymph nodes during the course of mannan-induced arthritis. (e) Correlation between the frequency of NKT1 (left panel), NKT2 (middle panel) and NKT17 (right panel) cells in joint draining lymph nodes and clinical arthritis score. (f) Expression of IFNγ by splenic NK cells 2, 4 and 6h after injection of LPS in WT BALB/c (blue) or SKG mice (red).(g.h.) Percentage (g) and cell numbers (f) of NKT1 ($PLZF^{lo}$ $ROR\gamma t^{-}$), NKT2 ($PLZF^{hi}$ $ROR\gamma t^{-}$) and NKT17 ($PLZF^{int}ROR\gamma t^{+}$) in the lymph nodes of WT and SKG mice. Representative of two independent experiments are shown. Graphs represent mean ± SEM with symbols representing individual mice. *P<0.05, **P<0.01, ***P<0.01 (One way ANOVA (a, b, d), Spearman Correlation test (a, e)), unpaired two-tailed Student's t test (f, g,h))

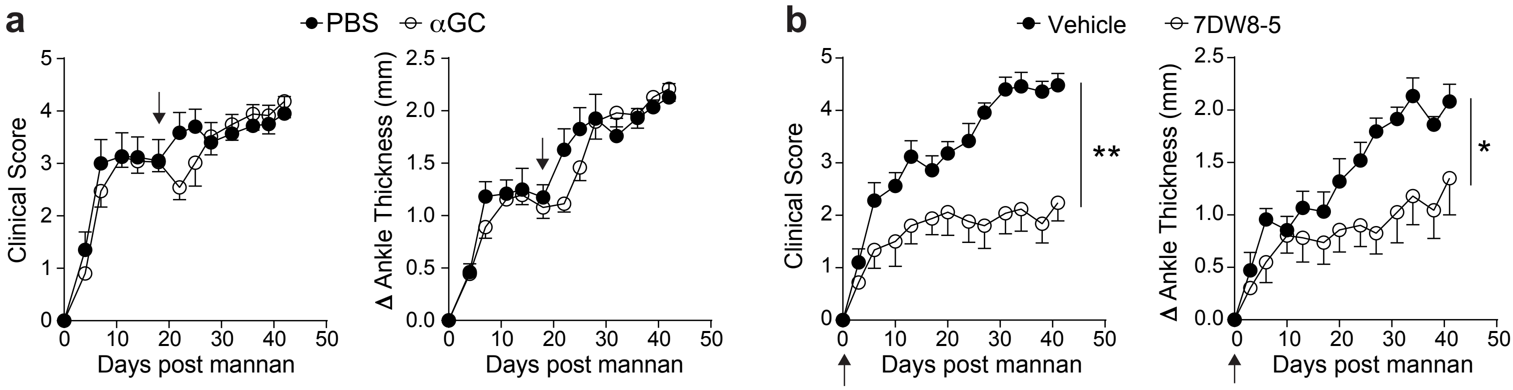

Supplementary Figure 10. Activation of iNKT cells affects the course of arthritis in SKG mice. (related to Figure 7)
(a) Clinical score (left panel) and change in ankle thickness (right panel) in SKG mice with mannan-induced arthritis treated with either PBS (i.p., n=6) or αGC (4 μg i.p., n=7) 17 days after mannan injection (treatment indicated by arrow). (b) Clinical score (left panel) and change in ankle thickness (right panel) in SKG mice with mannan-induced arthritis treated with either vehicle control (i.p., n=5) or the glycolipid antigen 7DW8-5 (4 ug, i.p., n=5) at the same day as arthritis induction (treatment indicated by arrow).
Compiled data from at least three independent experiments are shown. *P<0.05, **P<0.01 (Mann-Whitney on the area under curve).

RA Patients	
Number of patients	8
Gender (M/F)	4/4
Age (years)	56.5 ± 9.2
Disease duration (years)	6.1 ± 2.3
ESR (mm/h)	44.5 ± 20.8
CRP (mg/dl)	6.3 ± 3.1
Swollen Joint Count	9.1 ± 4.5
MTX treatment	5
GC treatment	1
aTNF treatment	2

Supplemental Table 1. RA patients characteristics (related to Fig. 6e-g).
ESR: Erythrocyte sedimentation rate,
CRP:C-reactive protein, MTX: Methotrexate,
GC: glucocorticoid, aTNF: anti-TNFα biological
